# Maternal ART throughout gestation prevents caudate volume reductions in neonates who are HIV exposed but uninfected

**DOI:** 10.3389/fnins.2023.1085589

**Published:** 2023-03-09

**Authors:** Abdulmumin Ibrahim, Fleur L. Warton, Samantha Fry, Mark F. Cotton, Sandra W. Jacobson, Joseph L. Jacobson, Christopher D. Molteno, Francesca Little, Andre J. W. van der Kouwe, Barbara Laughton, Ernesta M. Meintjes, Martha J. Holmes

**Affiliations:** ^1^Division of Biomedical Engineering, Department of Human Biology, Faculty of Health Sciences, Biomedical Engineering Research Centre, University of Cape Town, Cape Town, South Africa; ^2^Neuroscience Institute, University of Cape Town, Cape Town, South Africa; ^3^Department of Paediatrics and Child Health and Tygerberg Children’s Hospital, Faculty of Medicine and Health Sciences, Family Centre for Research with Ubuntu, Stellenbosch University, Stellenbosch, South Africa; ^4^Department of Psychiatry and Behavioral Neurosciences, Wayne State University School of Medicine, Detroit, MI, United States; ^5^Department of Psychiatry and Mental Health, University of Cape Town, Cape Town, South Africa; ^6^Department of Statistical Sciences, University of Cape Town, Cape Town, South Africa; ^7^A.A. Martinos Center for Biomedical Imaging, Massachusetts General Hospital, Boston, MA, United States; ^8^Department of Radiology, Harvard Medical School, Boston, MA, United States; ^9^Cape Universities Body Imaging Centre, University of Cape Town, Cape Town, South Africa

**Keywords:** HIV exposure, antiretroviral therapy, neonate, magnetic resonance imaging, brain structure, neurodevelopment

## Abstract

**Introduction:**

Successful programmes for prevention of vertical HIV transmission have reduced the risk of infant HIV infection in South Africa from 8% in 2008 to below 1% in 2018/2019, resulting in an increasing population of children exposed to HIV perinatally but who are uninfected (HEU). However, the long-term effects of HIV and antiretroviral treatment (ART) exposure on the developing brain are not well understood. Whereas children who are HEU perform better than their HIV-infected counterparts, they demonstrate greater neurodevelopmental delay than children who are HIV unexposed and uninfected (HUU), especially in resource-poor settings. Here we investigate subcortical volumetric differences related to HIV and ART exposure in neonates.

**Methods:**

We included 120 infants (59 girls; 79 HEU) born to healthy women with and without HIV infection in Cape Town, South Africa, where HIV sero-prevalence approaches 30%. Of the 79 HEU infants, 40 were exposed to ART throughout gestation (i.e., mothers initiated ART pre conception; HEU-pre), and 39 were exposed to ART for part of gestation (i.e., mothers initiated ART post conception; HEU-post). Post-conception mothers had a mean (± SD) gestational age (GA) of 15.4 (± 5.7) weeks at ART initiation. Mothers with HIV received standard care fixed drug combination ART (Tenofovir/Efavirenz/Emtricitabine). Infants were imaged unsedated on a 3T Skyra (Siemens, Erlangen, Germany) at mean GA equivalent of 41.5 (± 1.0) weeks. Selected regions (caudate, putamen, pallidum, thalamus, cerebellar hemispheres and vermis, and corpus callosum) were manually traced on T1-weighted images using Freeview.

**Results:**

HEU neonates had smaller left putamen volumes than HUU [β (SE) = −90.3 (45.3), *p* = 0.05] and caudate volume reductions that depended on ART exposure duration *in utero*. While the HEU-pre group demonstrated no caudate volume reductions compared to HUU, the HEU-post group had smaller caudate volumes bilaterally [β (SE) = −145.5 (45.1), *p* = 0.002, and −135.7 (49.7), *p* = 0.008 for left and right caudate, respectively].

**Discussion:**

These findings from the first postnatal month suggest that maternal ART throughout gestation is protective to the caudate nuclei. In contrast, left putamens were smaller across all HEU newborns, despite maternal ART.

## Introduction

Global preventative efforts have led to reduced perinatal transmission of the human immunodeficiency virus (HIV). In South Africa, successful programmes for prevention of vertical transmission have reduced the risk of perinatal transmission from 8% in 2008 to about 1.4% in 2016 and below 1% in 2018/2019 (National Department of Health, 2019), thereby preventing approximately 80,000–85,000 new vertical infections per annum (SAMRC, 2016). Consequently, there is an increasing population of infants who were exposed to HIV perinatally but are uninfected (HEU) in whom the long-term implications of *in utero* HIV and antiretroviral treatment (ART) exposure are not well understood. While perinatal HIV and ART exposure are not as damaging as HIV infection to the developing infant, exposure-related developmental delays and damage have nonetheless been reported (Le Roux et al., 2018; McHenry et al., 2018, 2019; Alcaide et al., 2019; Wedderburn et al., 2019b).

Maternal HIV likely contributes to developmental delays in infants and children who are HEU through changes to the mother’s immune system during pregnancy (Abu-Raya et al., 2016). Moreover, although ART improves maternal immune health by suppressing viral replication and enabling increased production of CD4 T cells (Volberding and Deeks, 2010) it may also be neurotoxic to the fetus (Abu-Raya et al., 2016; Lanman et al., 2021). Human and animal models provide evidence that maternal viral infections influence fetal and infant brain development (Ellman et al., 2009; Visentin et al., 2012; Parboosing et al., 2013; Bauman et al., 2014; Salemi et al., 2014; Choi et al., 2016), and an increased inflammatory response has been posited as a possible mechanism (Sappenfield et al., 2013). As it is still not well understood how viruses–including HIV–affect the developing fetus, it is important to monitor brain maturation in infants who are HEU (Wedderburn et al., 2019a).

The period between the second trimester of pregnancy and the first two years of postnatal life is critical for brain development (Alimenti et al., 2006). With magnetic resonance imaging (MRI) we are able to study the central nervous system (CNS) non-invasively. To date, twelve studies have examined effects of HIV exposure using MR brain imaging (Cortey et al., 1994; Tardieu et al., 2005; Jahanshad et al., 2015; Tran et al., 2016; Holmes et al., 2017; Jankiewicz et al., 2017; Robertson et al., 2018; Graham et al., 2020; Yadav et al., 2020; Bertran-Cobo et al., 2022; Madzime et al., 2022; Wedderburn et al., 2022a), but only three have included infants (age <1 year) who are HEU (Cortey et al., 1994; Tran et al., 2016; Wedderburn et al., 2022a)–one using structural MRI to examine volume differences between 40 HEU and 106 unexposed 2- to 4-week-old neonates (Wedderburn et al., 2022a), another using diffusion tensor imaging (DTI) to examine white matter integrity in 15 HEU neonates compared to 22 unexposed controls (Tran et al., 2016), and the third using MR spectroscopy to assess metabolism in 5 HEU newborns compared to 5 controls (Cortey et al., 1994).

Whereas some of the imaging studies referred to above found differences in regional volumes, localized metabolite levels and white matter integrity across different brain regions in infants and children who are HEU compared to their counterparts who are HIV unexposed and uninfected (HUU) (Cortey et al., 1994; Tardieu et al., 2005; Tran et al., 2016; Jankiewicz et al., 2017; Robertson et al., 2018; Graham et al., 2020; Yadav et al., 2020; Madzime et al., 2022; Wedderburn et al., 2022a), others did not find any HIV exposure-related differences (Jahanshad et al., 2015; Holmes et al., 2017). These discrepancies may be due to small sample sizes, differing imaging modalities and different ages of assessment. Notably, only one of these studies considered ART exposure duration relative to brain changes (Wedderburn et al., 2022a).

Non-imaging studies have also reported deficits in at least one neuropsychological domain in infants and children who are HEU compared to HUU (Forehand et al., 1998; Dorsey et al., 1999; Esposito et al., 1999; Sanmaneechai et al., 2005; Van Rie et al., 2009; Chaudhury et al., 2017; Familiar et al., 2018; Jao et al., 2020). A comprehensive review of the literature regarding early neurodevelopment (birth to 5 years) of children who are HEU was recently published by Wedderburn et al. (2022b). Specifically in Southern Africa, three studies, one each from South Africa (Wedderburn et al., 2019b), Botswana (Chaudhury et al., 2017) and Zimbabwe (Ntozini et al., 2020), reported language delay in HEU infants and children; two studies, one in South Africa (Le Roux et al., 2018) and one in Zimbabwe (Ntozini et al., 2020), poorer motor development; and one study (Springer et al., 2012) poorer adaptive behavior. Another study performed in South Africa found delayed gross and fine motor development (that was independent of ART initiation timing) in a higher proportion of HEU infants at age 12–24 months than typically seen in unexposed infants from similar communities (Madlala et al., 2020).

In the current study, we imaged neonates born to mothers living with and without HIV from the same community to examine HIV exposure-related changes in manually traced volumes of select subcortical structures and the cerebellum. Among the mothers living with HIV, approximately half had initiated treatment before conception and the others after conception, permitting us to examine effects of ART exposure duration. Imaging newborns provides insight into brain development highly related to the prenatal maternal environment while limiting the impact of the sub-optimal postnatal environment into which HEU children are often born. The only two previous studies to examine HIV exposure-related structural brain changes both used automated segmentation–one was conducted in infants and reported smaller caudate volumes bilaterally in HEU compared to HUU (Wedderburn et al., 2022a). The other was conducted in 10-year-old children and did not find any volumetric differences using tensor-based morphometry (Jahanshad et al., 2015). In view of the previously reported effects of prenatal HIV exposure on gross and fine motor movement (Le Roux et al., 2018; Madlala et al., 2020; Ntozini et al., 2020) and the recognized role of the basal ganglia and cerebellum in motor control and coordination, we hypothesized smaller volumes in these regions in HEU neonates compared to HUU. Since no effect of ART initiation timing was previously seen on gross and fine motor function, we hypothesized that volumes would not be impacted by duration of *in utero* ART exposure.

## Materials and methods

### Participants

**FIGURE 1:**
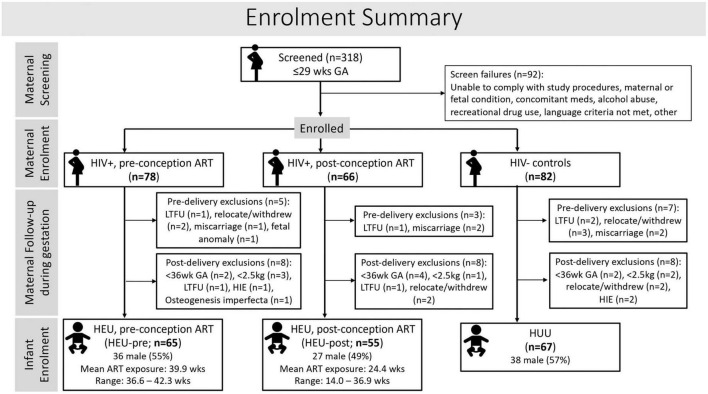
Flow diagram showing maternal and infant participant and exclusion numbers at each stage of the study.

Between 2017 and 2021, 226 Xhosa women (144 living with HIV; 82 uninfected controls), 18 years or older, with low-risk pregnancies were recruited at ≤29 weeks of gestation from community antenatal clinics in Cape Town, South Africa where HIV sero-prevalence approaches 30% (SANAC, 2018). Of the pregnant women living with HIV (PWLH), 78 had initiated ART before conception, exposing the fetus to antiretrovirals (ARVs) for the entire pregnancy, while 66 started ART after conception of the current pregnancy (Figure 1).

Maternal study exclusion criteria were underlying chronic disorders (e.g., diabetes, epilepsy, tuberculosis, hypertension), poor obstetric history (e.g., any previous second trimester miscarriage, stillbirth, neonatal deaths, hypertension, gestational diabetes, previous premature delivery), active tuberculosis or a known tuberculosis contact, current pregnancy related medical conditions (e.g., hypertension or diabetes); medication other than required pregnancy supplements (ferrous sulphate, folic acid, calcium carbonate) or ART, cotrimoxazole or isoniazid; among women with HIV, poor adherence to ART, non-standard ART regimens or non-disclosure of HIV status to family members; alcohol consumption around conception and/or during the current pregnancy of ≥7 drinks per week or ≥4 drinks per occasion; illicit drug use, or language criteria (Xhosa- or English-speaking) not met.

All women enrolled provided written informed consent in person in their preferred language before enrolling in the study. The study was conducted according to protocols that had been approved by the Health Sciences Human Research Ethics Committees of Stellenbosch University (M16/10/041) and the University of Cape Town (UCT; 801/2016). Mothers also provided consent for their infants to participate in the study.

At enrollment demographic information was recorded. Women had monthly study visits to coincide with planned routine antenatal clinic visits at the same site. Study visits included health monitoring (co-infections and concomitant medications), Harvard Trauma Scale (Halepota and Wasif, 2001) and Centre for Epidemiological Studies depression questionnaires (Roberts and Vernon, 1983; Pretorius, 1991). Mothers were also interviewed at each clinic visit regarding their smoking (cigarettes/day), alcohol and drug use using the timeline follow-back approach (Jacobson et al., 2002, 2008). Urine was tested for recreational drug use (cannabis, methamphetamine, and methaqualone) at study visits closest to 20 and 33 weeks of gestation. Gestational age (GA) was estimated by a combination of date of last menstrual period, fundal height, and early antenatal ultrasound performed at the clinic. Following delivery, the GAs at antenatal time points were adjusted according to the GA estimate at birth, taking into consideration the above factors and repeat ultrasound examination.

For women living with HIV, viral load (VL) and CD4 counts within 6 months of pregnancy and delivery were obtained from clinic records. As per standard care for all pregnant women in South Africa, HIV status is confirmed at the antenatal clinic most commonly by means of HIV Rapid test. VL and CD4 counts are measured yearly in previously diagnosed PWLH, and during pregnancy for those newly diagnosed. Since VL is only measured a couple of weeks after starting ART in newly diagnosed mothers, no VL data from the pre-ART period were available for mothers in the post-conception group. Only PWLH on fixed drug combination ART (Tenofovir/Efavirenz/Emtricitabine) were included in the study. An ART adherence questionnaire was administered at each study visit by an adherence counselor.

As per standard care, infants born to PWLH were given Nevirapine if considered low risk, with Zidovudine added if at high risk of vertical transmission of HIV. Infants are considered at high risk if maternal VL > 1000 copies/mL at 32 weeks gestation.

Infant exclusionary criteria were preterm delivery <36 weeks GA, neonatal hospital admission, birth weight <2500 g, positive on HIV-1 PCR, or conditions that could influence neurodevelopmental outcomes, such as severe congenital malformations or chromosomal abnormalities, neonatal asphyxia, persistent hypoglycemia, or severe neonatal jaundice. A summary of maternal enrollment and details of pre- and post-delivery exclusions is provided in Figure 1.

After exclusions, 187 mother-infant pairs were enrolled in the study, of whom 67 infants were HUU, 65 HEU with ART exposure throughout gestation (HEU-pre), and 55 HEU but exposed to ART for only part of their gestational period (HEU-post).

### MR data acquisition

Newborns and their mothers were transported to the Cape Universities Body Imaging Centre (CUBIC) located adjacent to the UCT Faculty of Health Sciences in Cape Town, South Africa, for MRI at a mean GA equivalent of 41.6 weeks (range 39–45 weeks). A pediatrician, blind to HIV and ART exposure status, weighed and examined the infant, and administered the Dubowitz Infant Neurological Examination (Dubowitz et al., 2005) about 1 h before the scheduled scan, following procedures developed in our previous newborn neuroimaging study (Jacobson et al., 2017). Infants were then fed, had their diaper changed, sponge earplugs inserted in their ears which were then covered with MiniMuffs^®^ (Natus Medical Incorporated, Middleton, WI, USA) and a beanie, and a pulse oximeter attached to one of their feet to monitor oxygen saturation. They were tightly swaddled, put to sleep supine on an MRI-compatible vacuum cushion containing styrofoam beads (VacFix,^®^ S&S Par Scientific, Houston, TX, USA) in the Siemens 16-channel pediatric head and neck coil, and imaged without sedation on a 3 T Skyra MRI (Siemens, Erlangen, Germany). The protocol included a high-resolution T1-weighted 3D echo-planar imaging (EPI) navigated multi echo magnetization prepared rapid gradient echo (MEMPRAGE) acquisition (FOV 192 × 192 mm^2^, TR 2540 ms, TI 1450 ms, TE’s = 1.69/3.55/5.41/7.27 ms, bandwidth 650 Hz/px, 144 sagittal slices, 1.0 × 1.0 × 1.0 mm^3^; GRAPPA factor 2) (van der Kouwe et al., 2008; Tisdall et al., 2012).

Of the 187 enrolled infants, 185 were seen at CUBIC (2 infants in the HEU-pre group missed their visit) and 166 (58 HEU-pre; 49 HEU-post; 59 HUU) provided imaging data. Scans were visually inspected for image quality and a subset of 120 high-quality scans used for manual tracing.

### Manual segmentation of target ROIs

**FIGURE 2:**
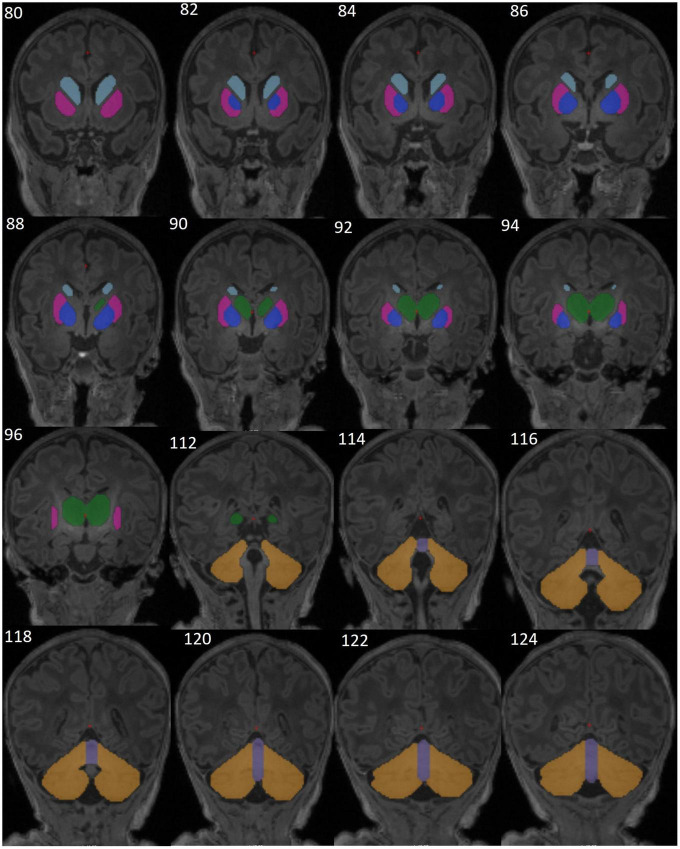
Representative T1-weighted brain MR images from one neonate without HIV exposure or infection (HUU), showing the manually segmented structures of the basal ganglia [caudate (sky blue), putamen (magenta), pallidum (dark blue)], thalamus (green), cerebellar hemispheres (yellow), and cerebellar vermis (purple). Images are in coronal view (slices 80-96 and 112-124).

The caudate nucleus, putamen, and globus pallidus of the basal ganglia, thalamus, cerebellar hemispheres, and cerebellar vermis (Figure 2) were manually traced using Freeview software (FreeSurfer v7.1.0 image analysis suite)^1^ on a Lenovo ThinkPad Yoga370 tablet. The contour of each structure was manually traced in the coronal plane and corrected on axial and sagittal planes on a slice-by-slice basis by a single expert neuroanatomist (AI). For the corpus callosum (CC) the images were first rotated around the anterior commissure-posterior commissure. The CC was then traced (by FW) in two contiguous midline sagittal slices in which the cerebral aqueduct was clearly visible and the areas of the two slices were averaged to obtain one representative value. The tracers were blinded to participants’ exposure status. Traced volumes were visually checked and verified by a senior neuroanatomist experienced in manual tracing (FW). To assess intra-rater reliability, a random subset of 10 brains were re-traced and intraclass correlation coefficients (ICC) for both consistency and absolute agreement computed for each region. Total brain volume for each subject was calculated using the infant FreeSurfer pipeline.

### Statistical analysis

All statistical analyses were performed in R statistical software (R Core Team, 2022). Student *t*-test/ANOVA and chi-square test for continuous and categorical variables, respectively, were used to compare sample characteristics between unexposed and HEU groups.

We used linear regression models to examine volumetric group differences: HUU vs. HEU, HUU vs. HEU-pre, and HUU vs. HEU-post. To determine the most salient confounders for each model, we calculated Pearson correlation coefficients between potential confounders and outcome volumes. Point biserial correlations were calculated for categorical variables. Possible confounders included four maternal indices [age at delivery, weight change per week, ounces absolute alcohol consumed per day averaged across pregnancy (oz AA/day), and education (highest grade completed)], infant sex and three infant indices at scan (GA equivalent, weight, and head circumference). Head circumference was used instead of total brain volume as it showed more and stronger associations with regional volumes. Potential confounders related at *p* < 0.10 to any regional volume were included in regression models. Pearson correlation and linear regression were also used to examine associations of regional brain volumes among HEU infants with maternal clinical and treatment variables. As the peak maternal VL during pregnancy variable was skewed, these data were log-transformed; mothers for whom no detectable VL (≥20 copies/mL) was ever measured during pregnancy were assigned a peak VL of 19 copies/mL. Since only a subset of mothers had detectable VL measurements during pregnancy, we also examined volumetric group differences between infants born to mothers whose VL measurements were always below detectable levels and those whose mothers had a detectable VL at least once during pregnancy. For the purposes of comparing the gestational periods with detectable VL (or gestational periods before the first undetectable VL) between HEU groups, and in view of the fact that no VL data were available from the period before initiating ART for mothers in the post-conception group, we assumed that the VL of mothers in the post-conception group was detectable until their first undetectable (< 20 copies/mL) VL measurement.

## Results

We present results for 120 infants (79 HEU; mean GA equivalent at scan ± standard deviation = 41.5 ± 1.0 weeks; 59 female). Of the 79 HEU infants, 40 were exposed to ART throughout gestation (HEU-pre) and 39 for only part of their gestational period (HEU-post). Mothers in the post-conception group initiated ART at a mean GA of 15.4 (±5.7) weeks. Sample characteristics are summarized in Table 1. Overall, maternal and infant indices were similar across groups, except that mothers in the HIV pre-conception group were about 3 years older than their post-conception and HIV-negative counterparts. Mothers in the HIV post-conception group gained less weight per week of pregnancy than mothers in the pre-conception group and those in the HUU group. Not surprisingly, mothers in the HIV pre-conception group had higher CD4 cell counts than those in the post-conception group. The mean gestational period during which VL was detectable was greater in the post-conception group. More women in the post-conception group had a detectable VL throughout pregnancy, and for those in whom VL did not remain detectable, the mean gestational period until first undetectable VL was longer in the post-conception group. There were only 10 mothers with positive drug tests–4 for cannabis (3 HUU; 1 HEU-pre), 5 for methamphetamine (3 HUU; 2 HEU-post) and 1 for methaqualone in the HUU group. Only 1 HUU mother reported use of tobacco. In contrast to the virtual absence of drug use and smoking, roughly 50% of mothers across groups reported drinking during pregnancy, albeit at very low levels (all < 0.10 oz AA/day).

**TABLE 1:** Sample characteristics (*N* = 120).

	HUU (*n* = 41)		HEU (*n* = 79)					
			**Pre-conception ART (*n* = 40)**		**Post-conception ART (*n* = 39)**			
	**Mean ± SD**	**Range**	**Mean ± SD**	**Range**	**Mean ± SD**	**Range**	**X^2^ or *t* or *F***	***P***
Age at delivery (years)	28.2 ± 5.7	19.6–44.1	31.4 ± 5.4	20.6–46.3	28.5 ± 4.8	19.6–40.9	4.45	**0.01**
Adjusted GA at enrolment (weeks)^1^	19.7 ± 5.5	8.1–28.0	20.9 ± 6.1	7.1–35.1	21.3 ± 6.1	9.1–30.7	0.83	0.44
Weight change across pregnancy (kg/week)	0.42 ± 0.22	−0.05–1.01	0.35 ± 0.20	−0.13–0.87	0.26 ± 0.23	−0.25–0.84	5.77	**0.004**
Highest school level completed (*n*,%)							11.47^a^	0.32
Grade 6	0 (0)	–	1 (2.5%)	–	0 (0)	–		
Grade 8	2 (4.9%)	–	1 (2.5%)	–	0 (0)	–		
Grade 9	0 (0)	–	3 (7.5%)	–	1 (2.6%)	–		
Grade 10	1 (2.4%)	–	3 (7.5%)	–	4 (10.3%)	–		
Grade 11	12 (29.3%)	–	15 (37.5%)	–	14 (35.9%)	–		
Grade 12	26 (63.4%)	–	17 (42.5%)	–	20 (51.3%)	–		
CD4 within 6 mo of pregnancy (cells/μL)^2^	N/A	N/A	573 ± 181	108–1025	434 ± 198	52–913	3.13	**0.003**
Detectable VL (≥20 copies/mL) at least once during pregnancy (*n*,%)	N/A	N/A	14 (35%)		17 (43.6%)		0.61	0.434
Peak VL during pregnancy (median [IQR])^3,4^	N/A	N/A	100 [33–1857]		398 [74–2439]		78.5^b^	0.183
Distribution of peak VLs during pregnancy (*n*,%)^4^							1.83^a^	0.151
<20 copies/mL	N/A	N/A	26 (65.0%)		22 (56.4%)			
≥20–400 copies/mL	N/A	N/A	9 (22.5%)		9 (23.1%)			
≥400–1000 copies/mL	N/A	N/A	1 (2.5%)		3 (7.7%)			
≥1000 copies/mL	N/A	N/A	3 (7.5%)		5 (12.8%)			
Gestational period with VL > 400 copies/mL (weeks)^5^	N/A	N/A	21.4 ± 13.7	3.4–32.6	34.1 ± 7.1	21.0–41.9	2.18	0.054
Gestational period with detectable VL (weeks)^6^	N/A	N/A	8.4 ± 13.0	0.0–41.6	31.9 ± 6.7	19.1–41.9	10.07	**<0.001**
Gestational period before first undetectable VL (weeks)^6,7^	N/A	N/A	5.5 ± 10.6	0.0–31.4	28.9 ± 5.5	19.1–39.0	10.71	**<0.001**
Detectable VL throughout pregnancy (*n*,%)	N/A	N/A	2 (5.0%)		11 (28.2%)		7.74	**0.005**
Substance use across pregnancy								
Alcohol (*n*;%)	20 (48.8%)	–	18 (45.0%)	–	22 (56.4%)	–	1.07	0.59
AA/day (oz)^8^	0.04 ± 0.03	0.057 × 10^–3^–0.096	0.03 ± 0.02	0.012–0.072	0.04 ± 0.02	0.009–0.072	0.79	0.46
Cannabis (*n*;%)^9^	3 (7.3%)	–	1 (2.5%)	–	0 (0)	–	3.45	0.18
Methamphetamine (*n*;%)^9^	3 (7.3%)	–	0 (0)	–	2 (5.1%)	–	2.85	0.24
Methaqualone (*n*;%)^9^	1 (2.4%)	–	0 (0)	–	0 (0)	–	1.94	0.38
Smoking (*n*;%)	1 (2.4%)	–	0 (0)	–	0 (0)	–	1.94	0.38
**Infant indices**								
Sex (*n* female;%)	21 (51.2%)	–	19 (47.5%)	–	19 (48.7%)	–	0.12	0.94
Delivery route							3.84	0.15
Vaginal (*n*,%)	35 (85.4%)	–	30 (75.0%)		26 (66.7%)	–		
Cesarean (*n*,%)	6 (14.6%)	–	10 (25.0%)		13 (33.3%)	–		
**Birth indices**								
GA (weeks)	39.8 ± 1.2	36.7–42.1	39.7 ± 1.4	36.6–42.1	39.7 ± 1.5	36.9–42.3	0.04	0.96
Weight (g)	3265 ± 388	2575–4180	3235 ± 347	2575–4255	3241 ± 387	2500–4230	0.07	0.93
Crown-to-heel length (cm)	49.9 ± 2.7	45.0–58.0	49.6 ± 2.2	44.0–54.0	50.1 ± 2.8	43.0–56.0	0.29	0.75
Head circumference (cm)	34.1 ± 1.2	32–37	34.0 ± 1.2	31–36	33.5 ± 1.5	31–39	2.09	0.13
ART exposure length (weeks)	N/A	N/A	39.7 ± 1.4	36.6–42.1	24.6 ± 5.5	14.0–35.6	16.1	**<0.001**
**MRI indices**								
GA Equivalent (weeks)	41.6 ± 0.9	40.0–43.9	41.3 ± 1.0	39.0–44.4	41.5 ± 1.2	39.0–43.4	0.50	0.61
Weight (g)^10^	3491 ± 405	2500–4250	3440 ± 421	2700–4500	3465 ± 397	2750–4450	0.15	0.86
Head circumference (cm)^10^	35.0 ± 1.3	32–37	35.5 ± 1.3	33–37	34.9 ± 1.2	31–36.5	2.48	0.09
Total brain volume (x 10^5^mm^3^)	3.64 ± 0.35	3.05–4.51	3.71 ± 0.32	2.99–4.53	3.64 ± 0.35	2.99–4.17	0.98	0.38

VL < 20 copies/mL undetectable; ^1^Following delivery, the GAs at antenatal timepoints were adjusted according to the GA estimate at birth; ^2^CD4 count missing for 5 mothers who started ART pre conception; ^3^Based only on mothers who had a detectable VL at least once during pregnancy; ^4^VL value missing for 1 pre-conception mother with detectable VL; ^5^Based only on mothers with VL ≥ 400 copies/mL at any point during pregnancy (4 mothers who started ART pre conception; 8 mothers who started ART post conception); ^6^Assuming VL of mothers in the post-conception group was detectable until their first undetectable measurement; ^7^Excluding 2 pre- and 11 post-conception mothers who never had undetectable VL measurements during pregnancy; ^8^Based only on mothers who consumed alcohol; 1 oz absolute alcohol (AA) is equivalent to 2 standard drinks; ^9^Numbers based on urine tests; ^10^Data missing for 1 infant in the pre-conception group and 2 HUU infants.

Bold indicates significance at *p* ≤ 0.05.

^a^Fisher Exact Test; ^b^Mann–Whitney U test.

In 10 brains that were retraced for intra-rater reliability assessment, ICCs for both absolute agreement and consistency were greater than 0.86 (all *p*’s < 0.002) for all traced regions.

**FIGURE 3:**
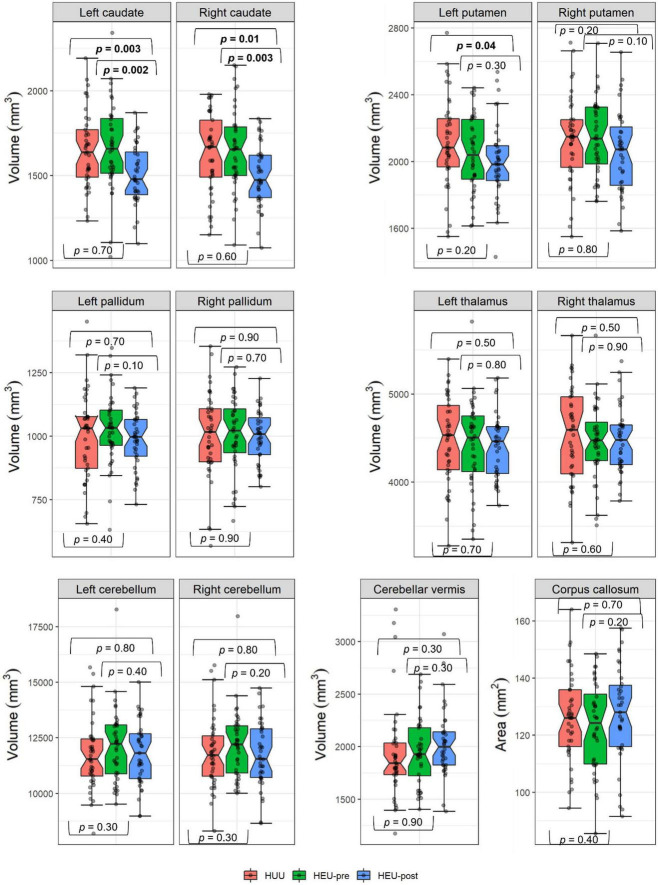
Comparison of regional volumes by group. The HEU group comprised infants who were HIV-exposed but uninfected born to mothers who had either initiated ART pre-conception (HEU-pre) or during gestation (post-conception; HEU-post); HUU are infants who were not HIV-exposed or infected. Hourglasses show median and interquartile ranges; whiskers are upper and lower extremes. Values above and below the whiskers are outliers, defined as data points more than 1.5 times the interquartile range above (or below) the upper (or lower) quartile. *p* values from independent one-tail student *t*-tests.

Figure 3 shows regional volumes by group, together with results from pairwise group comparisons using independent 1-tailed Student *t*-tests. Associations of potential confounders with regional volumes are summarized in Table 2. Infant sex, maternal age at delivery and maternal education were not associated with volumes in any regions.

**TABLE 2:** Associations of regional volumes with potential confounding variables.

ROI	Infant indices at MRI				Maternal indices			
	**Sex^1^**	**GA equivalent**	**Weight^2^**	**Head circumference^2^**	**Weight change per week**	**Age at delivery**	**AA/day**	**Education**
L caudate	−0.08 (0.37)	0.05 (0.55)	**0.21 (0.02)**	0.14 (0.13)	−0.06 (0.50)	−0.04 (0.64)	−0.15 (0.10)	0.03 (0.74)
R caudate	−0.04 (0.69)	−0.01 (0.95)	**0.18 (0.05)**	0.10 (0.28)	−0.03 (0.74)	0.004 (0.96)	−0.13 (0.15)	0.05 (0.59)
L putamen	0.02 (0.80)	0.09 (0.35)	**0.30 (<0.001)**	**0.20 (0.03)**	**0.16 (0.08)**	−0.01 (0.90)	−0.15 (0.10)	0.07 (0.46)
R putamen	0.04 (0.64)	0.09 (0.32)	**0.30 (<0.001)**	**0.24 (0.009)**	**0.16 (0.08)**	−0.02 (0.82)	−0.09 (0.32)	0.13 (0.17)
L pallidum	−0.05 (0.56)	−0.12 (0.19)	0.05 (0.53)	**0.21 (0.02)**	−0.02 (0.84)	−0.01 (0.93)	−**0.25 (0.005)**	0.14 (0.13)
R pallidum	−0.13 (0.17)	0.04 (0.70)	**0.17 (0.07)**	**0.16 (0.09**)	−0.03 (0.79)	−0.09 (0.33)	−**0.17 (0.06)**	0.15 (0.10)
L thalamus	−0.14 (0.13)	0.13 (0.15)	**0.38 (<0.001)**	0.09 (0.29)	−0.02 (0.86)	0.07 (0.44)	−0.06 (0.48)	0.07 (0.44)
R thalamus	−0.14 (0.14)	**0.16 (0.08)**	**0.38 (<0.001)**	0.09 (0.31)	−0.03 (0.77)	0.08 (0.38)	−0.01 (0.92)	0.09 (0.34)
L cerebellum	−0.09 (0.31)	**0.20 (0.03)**	**0.51 (<0.001)**	0.09 (0.35)	0.15 (0.11)	−0.03 (0.76)	−0.10 (0.27)	0.08 (0.38)
R cerebellum	−0.13 (0.14)	**0.20 (0.03)**	**0.48 (<0.001)**	0.07 (0.43)	0.15 (0.10)	−0.03 (0.76)	−0.13 (0.16)	0.11 (0.25)
Vermis	0.11 (0.23)	**0.17 (0.07)**	**0.31 (<0.001**)	−0.11 (0.24)	0.08 (0.39)	−0.09 (0.29)	0.03 (0.78)	0.04 (0.67)
Corpus callosum	−0.06 (0.52)	−0.06 (0.55)	0.12 (0.20)	**0.20 (0.03)**	−0.03 (0.74)	0.00 (0.99)	0.06 (0.49)	0.01 (0.90)

Values are Pearson correlation coefficients, r (*p*-value); ^1^For categorical variables, point biserial correlation was used; ^2^Data missing for 1 infant in the HEU pre-conception ART group and 2 HUU infants. GA, gestational age; AA, absolute alcohol (oz); L, left; R, right; Bold indicates significance at *p* < 0.10.

In Table 3 we present regression coefficients for the effect of group on regional volumes, controlling for potential confounding by covariates that are even weakly related to any volumetric outcome. As a group, HEU infants demonstrated smaller left putamen compared to HUU (mean ± SD; HEU = 2022 ± 229 mm^3^, HUU = 2115 ± 270 mm^3^). However, this difference fell below conventional levels of significance within each of the HEU sub-groups when compared separately to HUU. By contrast, smaller caudate nuclei were seen bilaterally in neonates in the HEU-post group compared to HUU (left caudate: HEU-post = 1508 ± 174 mm^3^, HUU = 1648 ± 227 mm^3^; right caudate: HEU-post = 1503 ± 183 mm^3^, HUU = 1626 ± 238 mm^3^). These reductions were *not* evident in the HEU-pre group (left caudate: HEU-pre = 1666 ± 253 mm^3^; right caudate: HEU-pre = 1650 ± 244 mm^3^, both *p*’s > 0.5). This is presumably why caudate reductions in the combined HEU group fell short of conventional levels of significance. Notably, the volume reduction in the left caudate in the HEU-post group remained significant after Bonferroni correction for comparisons in 12 regions.

**TABLE 3:** Regression coefficients for the effect of group on regional volumes, controlling for potential confounding by covariates at least weakly related to any volumetric outcome.

Region	HEU vs. HUU		HEU pre-conception ART vs. HUU		HEU post-conception ART vs. HUU	
	**β (SE)**	***p***	**β (SE)**	***p***	**β (SE)**	***p***
Left caudate	−73.9 (42.9)	0.09	−30.6 (53.6)	0.57	−145.5 (45.1)	**0.002**
Right caudate	−63.7 (45.0)	0.16	−16.9 (55.3)	0.76	−135.7 (49.7)	**0.008**
Left putamen	−90.3 (45.3)	**0.05**	−107.2 (55.9)	0.06	−86.8 (56.8)	0.13
Right putamen	−13.5 (45.0)	0.77	−0.6 (55.0)	0.99	−14.2 (55.1)	0.80
Left pallidum	7.0 (27.4)	0.80	22.6 (34.3)	0.51	−11.8 (34.0)	0.73
Right pallidum	2.3 (28.2)	0.94	−0.3 (36.7)	0.99	−6.2 (33.4)	0.85
Left thalamus	−67.1 (86.0)	0.44	−78.8 (109.6)	0.47	−83.7 (100.9)	0.41
Right thalamus	−95.5 (83.8)	0.26	−121.4 (107.7)	0.26	−90.7 (105.5)	0.39
Left cerebellum	387.5 (260.7)	0.14	356.9 (328.7)	0.28	335.2 (295.7)	0.26
Right cerebellum	264.8 (260.8)	0.31	278.8 (319.8)	0.39	160.0 (305.6)	0.60
Vermis	76.0 (71.4)	0.29	30.0 (89.9)	0.74	141.2 (89.8)	0.12
Corpus callosum	−1.1 (3.2)	0.72	−4.9 (3.6)	0.18	2.6 (3.8)	0.50

The model includes potential confounders related at least weakly (at *p* < 0.10) to any outcome: equivalent GA of infant at MRI; infant weight at MRI; infant head circumference at MRI; maternal weight change per week; maternal alcohol consumption averaged across pregnancy. Bold indicates significance at *p* ≤ 0.05. Infants who are HEU were compared to HUU initially as a combined group, and then stratified by ART exposure duration [ART throughout gestation (pre-conception ART) or for only part of gestation (post-conception ART)]. Values are unstandardized regression coefficients (β) and standard errors (SE).

**FIGURE 4:**
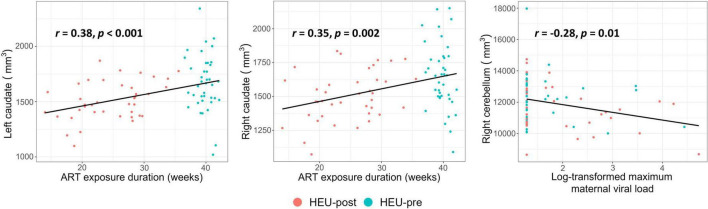
Plots showing associations of regional volumes with maternal clinical and treatment variables among infants who were HIV-exposed but uninfected. All associations shown remained significant after control for potential confounding variables.

In Table 4 and Figure 4 we present associations of regional volumes among HEU infants with maternal clinical and treatment variables. In both the left and right caudate, increasing duration of ART exposure was associated with increasing volume, effects that remained significant after control for potential confounding. Higher peak maternal VL during pregnancy was associated with smaller volumes of the left putamen, the cerebellar hemispheres and the vermis. This effect was still observed in the right cerebellar hemisphere after controlling for potential confounding variables (Figure 4).

**TABLE 4:** Associations of regional volumes with maternal clinical and treatment variables in infants with perinatal HIV/ART exposure.

Region	Maternal CD4 within 6 mo of pregnancy (*n* = 74)^a^		Peak maternal VL during pregnancy (*n* = 78)^b^		Infant ART exposure duration (*n* = 79)		Maternal VL during pregnancy: Detectable vs. Undetectable (*n* = 79)^c^	
	***r*** **(*p*)**	**β (*p*)**	***r*** **(*p*)**	**β (*p*)**	***r*** **(*p*)**	**β (*p*)**	***t*** **(*p*)**	***F*** **(*p*)**
Left caudate	0.21 (0.07)	0.17 (0.12)	−0.21 (0.06)	−0.18 (0.11)	**0.38 (<0.001)**	**8.60 (0.004)**	1.11 (0.27)	0.56 (0.48)
Right caudate	0.09 (0.45)	0.07 (0.57)	−0.20 (0.08)	−0.18 (0.12)	**0.35 (0.002)**	**8.45 (0.007)**	0.61 (0.34)	0.39 (0.54)
Left putamen	0.10 (0.39)	0.07 (0.50)	−**0.23 (0.04)**	−0.15 (0.15)	0.09 (0.44)	−0.58 (0.84)	1.75 (0.08)	1.28 (0.26)
Right putamen	0.12 (0.30)	0.12 (0.24)	−0.22 (0.06)	−0.14 (0.16)	0.14 (0.23)	2.26 (0.45)	1.31 (0.19)	0.56 (0.47)
Left pallidum	0.02 (0.89)	−0.03 (0.77)	−0.12 (0.27)	−0.14 (0.21)	0.13 (0.26)	0.92 (0.59)	1.13 (0.26)	1.57 (0.22)
Right pallidum	0.08 (0.95)	−0.02 (0.90)	−0.06 (0.57)	−0.04 (0.73)	0.04 (0.71)	−0.11 (0.95)	1.07 (0.29)	0.86 (0.36)
Left thalamus	0.00 (0.99)	−0.01 (0.95)	−0.16 (0.17)	−0.09 (0.43)	0.05 (0.68)	1.48 (0.80)	0.66 (0.51)	0.01 (0.94)
Right thalamus	−0.04 (0.72)	−0.04 (0.72)	−0.18 (0.11)	−0.11 (0.32)	0.01 (0.93)	−0.92 (0.86)	0.91 (0.36)	0.03 (0.86)
Left cerebellum	0.02 (0.85)	−0.00 (0.99)	−**0.24 (0.03)**	−0.15 (0.13)	0.11 (0.33)	9.29 (0.62)	1.19 (0.24)	0.13 (0.72)
Right cerebellum	0.04 (0.72)	0.01 (0.90)	−**0.28 (0.01)**	−**0.20 (0.05)**	0.14 (0.22)	13.61 (0.46)	1.26 (0.21)	0.25 (0.62)
Vermis	−0.02 (0.86)	−0.03 (0.77)	−**0.24 (0.03)**	−0.19 (0.09)	−0.08 (0.47)	−4.81 (0.28)	1.68 (0.10)	1.59 (0.21)
Corpus callosum	−0.07 (0.53)	−0.06 (0.63)	−0.09 (0.44)	−0.09 (0.48)	−0.08 (0.49)	−0.16 (0.50)	0.35 (0.73)	0.12 (0.73)

*r* is Pearson correlation coefficient; β is regression coefficient after controlling for potential confounders related to any of the volumetric outcomes at *p* < 0.10: equivalent GA of infant at MRI; infant weight at MRI; infant head circumference at MRI; maternal weight change per week; maternal alcohol consumption averaged across pregnancy. Bold indicates significance at *p* ≤ 0.05.

^a^CD4 count missing for 5 mothers who started ART pre conception; ^b^Using log-transformed viral load (VL) with undetectable peak VL recoded to VL = 19 copies/mL before transformation; VL value not available for 1 mother who started ART pre conception; ^c^Group comparisons including all HEU infants.

## Discussion

Using structural MRI in a cohort of newborns, we observed HIV exposure-related volume reductions in the left putamen and bilateral caudates. Specifically, left putamens were smaller by 4.4% in HEU than HUU, and left and right caudates by 8.5 and 7.6%, respectively, in the HEU-post group *only* compared to HUU. Since the reductions in caudate volumes were evident only in infants from the HEU-post group, maternal ART appears to protect the caudate of the developing fetal brain from HIV-related damage. This finding is further supported by the associations we found between caudate volumes and duration of prenatal ART exposure. The observed fetal neuroprotection may be related to maternal immune health, as maternal CD4 count was positively associated with infant left caudate volume, albeit below conventional levels of significance. Higher peak viral loads during pregnancy were, in addition, associated with reduced volumes of the left putamen, the cerebellar hemispheres and the vermis. The absence of associations in these regions with ART duration suggests that treatment from conception does not offer complete neuroprotection.

### Basal ganglia

Among the subcortical structures manually segmented, nuclei of the basal ganglia were affected by maternal HIV and ART. Of the imaging studies in children who are HEU that examined subcortical structures, one reported altered metabolism in the basal ganglia at age 9 years (Robertson et al., 2018) and a recent infant volumetric study that used automated segmentation in SPM reported reduced caudate and total gray matter volumes (Wedderburn et al., 2022a). In contrast to the current study, Wedderburn et al. found no relation between maternal ART initiation timing and infant global or regional brain volumes. This discrepancy may be due to the smaller sample size of that study which had only 40 infants who were HEU, of whom 62.5% were HEU-post (i.e., their mothers initiated ART post-conception). Overall, findings from our present study confirm those of that study, since the larger post-conception group in that study may have biased the results and may account for the observed caudate volume reductions. If the caudate volumes of the smaller HEU-pre group were not different from the HUU group, as seen in the current study, this would have diluted the group difference, accounting for the smaller volume reductions than seen in the HEU-post group here (left caudate 4.1 vs. 8.5%; right caudate 6.6 vs. 7.6%).

Within the basal ganglia, the caudate may be particularly vulnerable due to its close proximity to the ventricular system of the brain. It is hypothesized that certain debris, such as infected microphages and circulating leukocytes, penetrate the blood brain barrier (BBB) and pass into structures adjacent to the ventricles (Ances et al., 2012). The invasion of the caudate nuclei by these proinflammatory cytokines may lead to volume reductions *via* inflammation and gliosis (Mehta et al., 2003; Pekny and Pekna, 2016). Although the precursors of the basal ganglia, the lateral and medial ganglionic eminences, first appear at embryonic stage 14 (32 postovulatory days), the basal ganglia only assume their adult shape at embryonic stage 21 (around 52 postovulatory days). The caudate and the future nucleus accumbens area first appear at stage 19 (47–48 postovulatory days), and the putamen and pallidum at stage 20 (50–51 postovulatory days; Nunta-aree et al., 2001). Exposure to HIV during these early developmental stages in the HEU-post group may explain why the reductions in caudate volumes are seen in this group only.

Maternal infection and inflammation have also been shown to increase oxidative stress in the fetal brain (Dani et al., 2004; Oskvig et al., 2012; Talukdar et al., 2020). Laboratory studies have demonstrated alterations in neuronal and glial development following fetal oxidative stress (Lanté et al., 2007; Barron et al., 2021), and it has been suggested as a mechanism behind the damage associated with prenatal alcohol and stimulant exposure (Brocardo et al., 2011; Wells et al., 2016) and preterm birth (Back et al., 2005). The caudate and putamen may be particularly vulnerable to oxidative stress, as dopaminergic cell bodies in the substantia nigra pars compacta, which project to the striatum, have been shown to be highly susceptible to oxidative damage (Wang and Michaelis, 2010). Since the gestational period during which viral loads were detectable was longer in the mothers who initiated ART post conception, it is not surprising that the caudate nuclei, which are particularly vulnerable to prenatal insult (Mutch et al., 1993), of infants born to these mothers would have been more highly impacted by the effects of maternal infection.

In contrast, the HIV exposure-related reduction in left putamen volume is evident in both the HEU-pre and HEU-post groups, despite falling short of conventional levels of significance when each of the sub-groups are compared separately to HUU. The fact that this result is independent of maternal ART initiation timing suggests that damage to this region may be occurring later in pregnancy when maternal immune marker and viral profiles of the HEU-pre and HEU-post groups are more similar. Alternatively, since the putamen is not in direct contact with the ventricles, it may be less sensitive to subtle changes in maternal immune markers. Putamen volume has been shown to be lateralized in normally developing infants, although results are inconsistent as to the direction of asymmetry (Choe et al., 2013; Dean et al., 2018). Our finding of lower volumes of the left putamen in the HEU infants suggests that prenatal exposure to HIV may alter brain lateralization.

Altered basal ganglia volumes are frequently reported in children living with HIV and receiving treatment (Becker et al., 2011; Li et al., 2018). Li et al. (2018) reported reduced right pallidal volume in adolescents living with HIV, while Becker and colleagues reported reduced caudate and putamen volumes in the presence of ART in their studies of older adults. Both authors linked the reduced volumes to the time since infection and lower CD4 count. Interestingly, our finding of reduced caudate volumes in neonates who were exposed to HIV but are uninfected was associated with shorter ART exposure durations *in utero* and lower maternal CD4 counts.

The putamen forms part of the dorsal striatum in the basal ganglia, which is important for motor control but also plays roles in learning and executive function (Lanciego et al., 2012). The caudate, in addition to its primary roles in directed movement and spatial integration, is involved in executive function, memory, procedural and associative learning, and inhibitory control, and is part of the reward system (Leisman et al., 2014). Smaller putamen and basal ganglia in children have been linked with higher ADHD scores (Li et al., 2022), and poorer working memory (Pangelinan et al., 2011), cognition, academic achievement and motor function (Loh et al., 2020). Childhood caudate size has been associated with general cognitive abilities (Pangelinan et al., 2011) and executive function (Saito et al., 2022). Reductions in caudate and putamen volume in the neonatal period may therefore underpin the neurodevelopmental delays previously reported in HEU children (Kerr et al., 2014; Benki-Nugent et al., 2022). Interestingly, the left putamen has been shown to be involved in various aspects of language and semantic processing (Viñas-Guasch and Wu, 2017), with performance on aspects of verbal fluency predictive of left putamen volume (Thames et al., 2012). The current finding of smaller left putamen volumes in HEU infants thus supports studies which have shown poorer language development and functioning in HEU children (Alcock et al., 2016; Ntozini et al., 2020).

### Maternal HIV infection and the uninfected neonate brain

The volume reductions seen among HEU neonates demonstrate that maternal HIV infection, even with ART, still influences the uninfected infant brain. While ART provides some level of immune restoration, people living with HIV on treatment still experience higher levels of immune activation and inflammation (Wada et al., 2015). Proinflammatory cytokines secreted from maternal decidua immune cells are elevated in pregnant mothers living with HIV compared to their uninfected counterparts (Lee et al., 2001; Vyas et al., 2021). The increasing number of proinflammatory cytokines may disrupt immune equilibrium at the materno-fetal interface. Exposure to maternal infection likely impacts the fetal brain indirectly *via* maternal inflammation cascades at the level of placentation. Additionally, exposure to the maternal immune response likely has longer term consequences, with HEU infants demonstrating an increased frequency of activated T cells compared to HUU (Clerici et al., 2000; Vigano et al., 2007; Bunders et al., 2014). Therefore, volume reductions observed in our cohort of HEU infants may be a result of a maternal immune response rather than directly from fetal primary responses.

### Maternal ART initiation and the uninfected infant brain

Despite evidence of improved prevention of vertical transmission of HIV among mothers who initiated ART pre conception compared to those who started ART during pregnancy (Mandelbrot et al., 2015; Agabu et al., 2020), pre-conception ART has been linked with increased risk of still-births, preterm delivery (PTD), and small for gestational age or low birth weight infants (Chen et al., 2012; Uthman et al., 2017; Snijdewind et al., 2018). However, we are not aware of any studies that have reported negative effects of longer prenatal ART exposure on infant neurodevelopmental outcomes. In contrast to the poorer birth outcomes reported in the above studies, the numbers of miscarriages/fetal anomalies among mothers enrolled in our study were similar across groups, as were the numbers of infants excluded due to PTD, low birth weight or a medical diagnosis (8.0% HUU; 9.6% HEU-pre; 7.9% HEU-post). Moreover, all infant indices at birth were similar across groups. Discrepancies with previous studies may be attributable to optimized ART regimens in the current study; all women living with HIV were on a fixed drug combination ART (Tenofovir/Efavirenz/Emtricitabine).

To date, it is not clear what the optimal period and timing of ART treatment during pregnancy is to minimize HIV-related damage to the fetus. Since the development of neurons and glial cells (responsible for providing nutrients, support and protection) is accelerated during the first trimester when the foundation of brain structures and functions are laid (Stiles and Jernigan, 2010; Reemst et al., 2016; Tiwari et al., 2018), any disturbances over this period may have severe long-term consequences. Pre-conception ART may mitigate HIV-related neural damage by lowering maternal viral loads and preventing an aggressive aggravated maternal immune response (Maharaj et al., 2017). A study from Namibia assessing the nationwide effectiveness of ART in the prevention of vertical transmission of HIV reported the lowest transmission rates in mothers who started ART pre conception (0.78%) compared to those who started post conception (0.98%), after delivery (4.13%), or who did not receive ART at all (11.62%) (Agabu et al., 2020). Conversely, in women who started ART later during pregnancy, elevations of pro-inflammatory cytokines in the placenta and eventually in the fetal brain during early pregnancy may trigger or precipitate disruptions in brain development.

In this study, we profiled maternal CD4 cell count and viral loads during pregnancy as a measure of maternal immune health. Our observation of the association of increasing peak maternal viral load during pregnancy with decreasing volumes of several brain regions suggests that infants born to mothers living with more poorly controlled HIV are at greater risk for regional damage. Although we did not see volumetric group differences in the cerebellar hemispheres and vermis, effects of HIV exposure on these areas have been demonstrated in DTI studies which noted microstructural alterations in cerebellar white matter in infants and children with HEU (Tran et al., 2016; Yadav et al., 2020).

### Limitations

In this study we did not quantify migratory monocytes, which may be used to infer the extent of neuroinflammation. An upsurge in infected migratory monocytes is thought to initiate a pro-inflammatory pathway (Valcour et al., 2012; Anzinger et al., 2014). Additionally, the results reported here are cross-sectional, and we did not examine associations of brain volumes with functional domains, nor differences in white matter microstructure. Follow-up studies are needed to examine these aspects further.

## Conclusion

Despite maternal ART, HEU infants demonstrate smaller basal ganglia nuclei volumes compared to HUU. While maternal ART from conception provided neuroprotection for the bilateral caudate, the left putamen volume was reduced across all HEU newborns. Follow-up studies are warranted to examine whether volume reductions persist or resolve in childhood. Further study should consider quantifying migratory monocytes in these HEU strata–pre- and post-conception ART exposure–to identify the mechanisms driving the volumetric effects from variations in ART exposure duration.

## Data availability statement

The raw data supporting the conclusions of this article will be made available by the authors, without undue reservation.

## Ethics statement

This study, which involved human participants, was reviewed and approved by the Health Sciences Human Research Ethics Committee of Stellenbosch University and the Human Research Ethics Committee of the Faculty of Health Sciences, University of Cape Town. Written informed consent to participate in this study was provided by the mothers in this study for themselves and on behalf of their infants.

## Author contributions

AI: data analysis and interpretation of results, and drafting manuscript. FW: data analysis and interpretation of results, and drafting and review of manuscript. SF and CM: data acquisition. MC: conception and design of study. SJ: conception and design of data acquisition procedures and instruments, and review of manuscript. JJ: conception and design of data acquisition instruments. FL: data analysis and review of manuscript. AvdK: conception and design of study, study oversight, and design of data acquisition instruments. BL: conception and design of study, study oversight, data acquisition and analysis, interpretation of results, and review of manuscript. EM: conception and design of study, study oversight, data acquisition and analysis, interpretation of results, and drafting and review of manuscript. MH: oversight, data analysis, interpretation of results, and drafting and review of manuscript. All authors contributed to the article and approved the submitted version.

## Footnotes

1
http://surfer.nmr.mgh.harvard.edu/
